# Does the XPA–FEN1 Interaction Concern to Nucleotide Excision Repair or Beyond?

**DOI:** 10.3390/biom14070814

**Published:** 2024-07-09

**Authors:** Yuliya S. Krasikova, Ekaterina A. Maltseva, Svetlana N. Khodyreva, Alexey N. Evdokimov, Nadejda I. Rechkunova, Olga I. Lavrik

**Affiliations:** 1 Institute of Chemical Biology and Fundamental Medicine, 630090 Novosibirsk, Russia; y.krasikova@gmail.com (Y.S.K.); 060179@mail.ru (E.A.M.); svetakh@niboch.nsc.ru (S.N.K.); an_evdokimov@mail.ru (A.N.E.); nadyarec@niboch.nsc.ru (N.I.R.); 2 Department of Natural Sciences, Novosibirsk State University, 630090 Novosibirsk, Russia

**Keywords:** DNA repair, DNA replication, nucleotide excision repair, FEN1, XPA

## Abstract

Nucleotide excision repair (NER) is the most universal repair pathway, which removes a wide range of DNA helix-distorting lesions caused by chemical or physical agents. The final steps of this repair process are gap-filling repair synthesis and subsequent ligation. XPA is the central NER scaffolding protein factor and can be involved in post-incision NER stages. Replication machinery is loaded after the first incision of the damaged strand that is performed by the XPF–ERCC1 nuclease forming a damaged 5′-flap processed by the XPG endonuclease. Flap endonuclease I (FEN1) is a critical component of replication machinery and is absolutely indispensable for the maturation of newly synthesized strands. FEN1 also contributes to the long-patch pathway of base excision repair. Here, we use a set of DNA substrates containing a fluorescently labeled 5′-flap and different size gap to analyze possible repair factor–replication factor interactions. Ternary XPA–FEN1–DNA complexes with each tested DNA are detected. Furthermore, we demonstrate XPA–FEN1 complex formation in the absence of DNA due to protein–protein interaction. Functional assays reveal that XPA moderately inhibits FEN1 catalytic activity. Using fluorescently labeled XPA, formation of ternary RPA–XPA–FEN1 complex, where XPA accommodates FEN1 and RPA contacts simultaneously, can be proposed. We discuss possible functional roles of the XPA–FEN1 interaction in NER related DNA resynthesis and/or other DNA metabolic processes where XPA can be involved in the complex with FEN1.

## 1. Introduction

For more than a decade, a growing pool of data has suggested that all DNA metabolic pathways interact with each other to form a network for maintaining genome protection. In particular, in nucleotide excision repair (NER), resynthesis stage is created by replication machinery that is loaded on DNA after the first incision of the damaged strand [1,2]. This incision is performed by the XPF–ERCC1 nuclease on the 5′ side of the damage site with the formation of a free 3′-OH group [3]. The damaged flap is incised by the XPG after partial gap filling. Possible replication machinery compositions are “DNA polymerase δ/PCNA/RFC1-RFC-p66”, “DNA polymerase ε/CTF18/RFC”, or “DNA polymerase κ/ubiquitinated PCNA/XRCC1” [1,2,4]. A protein set composition depends on ubiquitination status of PCNA. One of PCNA’s protein partners is flap endonuclease 1 (FEN1). Structures of PCNA–FEN1 complexes as well as ternary complexes with other proteins and DNA were solved using X-ray and cryo-electron microscopy [5,6,7,8].

FEN1 is a highly conserved structure-specific nuclease that catalyzes a specific incision to remove 5′-flaps in duplex DNA substrates [9,10]. These kinds of DNA structures can arise in replication and repair processes of pro- and eukaryotes when a replication- or repair-associated polymerase displaces a downstream DNA segment into a 5′-flap structure. FEN1 is able to recognize and cleave DNA at the base of a 5′-flap, thereby effectively creating a nick that is sealed by DNA ligase 1. Similarly, in long-patch base excision repair (BER), if DNA polymerase β cannot remove the 5′-deoxyribose moiety for some reason, a DNA segment approximately 2–20 nucleotides in length is synthetized, displacing the downstream DNA into a 5′-flap, which is subsequently processed by FEN1 and DNA ligase 1 [11,12]. Thus, FEN1 is a critical component of replication machinery and is absolutely indispensable for newly synthesized strand maturation.

Besides its role in DNA replication and BER, FEN1 participates in other cellular pathways. Recent studies showed that FEN1 can be involved in R-loop resolution [13,14]. R-loops occur naturally during gene transcription when a nascent RNA strand hybridizes back to its DNA template [15]. It has been reported that FEN1 is recruited to R-loops in normal human fibroblasts [13,14]. Moreover, recent findings suggest that FEN1 can cleave a noncoding single-stranded DNA (ssDNA) strand of an R-loop to induce double-strand DNA (dsDNA) breaks in coordination with topoisomerase I and nucleases XPF–ERCC1 and XPG [16].

XPA is the central NER scaffolding protein factor that interacts with almost all NER participants and organizes the correct NER repair complex [17,18,19]. In the absence of XPA’s scaffolding function, no repair process occurs. We have shown previously that XPA can remain in the NER complex after the first incision via a protein–protein interaction with RPA, which is bound to the undamaged strand [20]. Thereafter, RPA recruits clamp loader RFC [21,22], and XPA possibly promotes the positioning of the PCNA clamp [23,24,25]. XPA and RPA also interact tightly inside the NER repair bubble [26,27], and together they regulate the correct orientation and the activation of NER nucleases XPG and XPF–ERCC1 [28,29,30,31,32,33,34,35].

Therefore, we hypothesize that FEN1 can reach an NER intermediate together with replication machinery during its recruitment by RPA and/or XPA. This hypothesis is supported by a study where FEN1 was found on an immobilized NER substrate [36]. In the present work, to further understand interplay between DNA replication and repair and reveal new possible ways within the interactome of these processes, we analyzed interactions between XPA, RPA, and FEN1 in the presence of DNA substrates imitating intermediates of the late stages of NER. We examined FEN1’s ability to cleave DNAs bearing a bulky lesion inside a 31 nt 5′-flap in combination with a 3, 10, or 26 nt gap. As expected, FEN1 effectively bonded to the DNAs and catalyzed flap incision. We detected formation of the XPA–FEN1–DNA ternary complex with each tested DNA. Moreover, we registered the XPA–FEN1 complex in the absence of DNA. Functional assays revealed that XPA slightly inhibits FEN1 catalytic activity at a high XPA concentration. Data on RPA–XPA–FEN1 complex formation where XPA accommodates FEN1 and RPA contacts simultaneously were obtained. Finally, we discuss functional roles of these complexes in the NER-related DNA resynthesis and other DNA processing pathways.

## 2. Materials and Methods

### 2.1. Reagents and Oligonucleotides

[γ-^32^P]ATP (3000 Ci/mmol) was synthesized in the Laboratory of Biotechnology (Institute of Chemical Biology and Fundamental Medicine, Novosibirsk, Russia). Phage T4 polynucleotide kinase was purchased from Biosan (Novosibirsk, Russia), BSA from Sigma, and stained molecular weight markers from Bio-Rad (Hercules, CA, USA). Reagents for electrophoresis and buffer components were acquired from either Sigma (Burlington, MA, USA) or Russian vendors (extra pure grade). All oligonucleotides were obtained commercially. Oligonucleotide Fg bearing a fluorescein dUMP derivative (5-{3-[6-(carboxyamido-fluoresceinyl)amidocapromoyl]allyl}-dUMP) (Flu-dUMP) was custom-synthesized by Nanotech-C (Russia). The DNA structures are presented in Table 1; the sequences of the oligonucleotides are given in Supplementary Materials (Table S1).

### 2.2. Protein Purification

Plasmid pET28-FSH (w_t.FE) bearing cDNA of human *FEN1* was kindly provided by Dr. R. Prasad (National Institute of Environmental Health Sciences, NC, USA). Recombinant human FEN1 was produced in an *Escherichia coli* system and purified essentially as described in [37] with some modifications. Briefly, *E. coli* strain BL21(DE3) transformed with plasmid pET28-FSH was grown at 30 °C, and synthesis of FEN-1 was induced with IPTG at a final concentration of 0.5 mM for 4 h at 25 °C. The cells were collected by centrifugation and lysed. DNA was fragmented by sonication followed by removal of cell debris. Recombinant human FEN1 (hFEN1) that contains a His-tag at the C-terminus was purified via several chromatographic steps: Ni-NTA agarose, SP-Sepharose, and heparin Sepharose chromatography. The obtained hFEN1 sample was >95% pure according to analysis by SDS polyacrylamide gel electrophoresis [38] and Coomassie staining. The purified enzyme was stored in a buffer (40 mM sodium phosphate buffer pH 7.8, 50% glycerol, 0.1 M NaCl, 0.1% of NP-40, 1 mM EDTA, and 5 mM DTT) at −20 °C.

Recombinant hXPA bearing an N-terminal His-tag was overexpressed in *E. coli* strain BL21(DE3)LysS, using the pETI5b-XPA recombinant plasmid kindly provided by Dr. O. Schärer (SUNY Stony Brook, NY, USA). The hXPA purification protocol was based on previously published procedures [39,40] with a modification of the order of chromatographic columns. The first column was Talon (Clontech, Mountain View, CA, USA) (instead of Ni-NTA), then a hydroxyapatite column (Bio-Rad), and finally a Superdex 75 gel filtration column (GE Healthcare, Chicago, IL, USA). The purified protein was aliquoted and stored at −70 °C.

Recombinant hRPA was purified from *E. coli* as described in [41]. The plasmid containing the cDNA of hRPA was a kind gift from Dr. M.S. Wold (Department of Biochemistry, Carver College of Medicine, University of Iowa, Iowa City, IA, USA).

### 2.3. Fluorescent Labeling of XPA

For XPA labeling, we used the method described previously [20,42] with modifications of protein separation from the reagents. The 5(6)-carboxyfluorescein N-hydroxysuccinimide ester (NHS ester) was dissolved in dimethyl sulfoxide to a final concentration of 20 mM. The reaction mixture (50 µL) contained 10 µL of 20 mM NHS ester and 10 µL of 15 µM XPA in buffer A (50 mM Tris-HCl pH 7.5, 100 mM KCl, 1 mM dithiothreitol). The reaction mixture was incubated overnight at 4 °C, and then conjugates were separated from the reagents by gel permeation chromatography on a Sephadex G-25 Superfine (GE Healthcare). The chromatography liquid phase consisted of buffer A. All collected fractions were analyzed by electrophoresis according to Laemmli [38] with subsequent fluorescence quantification using a gel documentation system (Amersham Imager 600, GE Healthcare) and Coomassie staining to quantify the efficiency of XPA labeling. The concentration and stoichiometry of fluorescein (FAM)–XPA conjugates (Flu–XPA) were determined on a POLARstar OPTIMA multifunctional microplate reader (BMG LABTECH GmbH, Ortenberg, Germany). The Flu-XPA concentration in the sample was used as total protein concentration.

### 2.4. Preparation of 5′-^32^P-Labeled Oligonucleotides

The radioactive label was attached to the 5′-end of oligonucleotides using phage T4 polynucleotide kinase as described before [43]. The labeled oligonucleotides were purified on MicroSpin^TM^ G-25 columns (GE Healthcare) or by electrophoresis under denaturing conditions followed by passive elution with 3 M LiClO_4_ and acetone precipitation. The concentration of the 5′-^32^P-labeled oligonucleotides was determined by electrophoresis under denaturing conditions using aliquots from the initial reaction mixture and from the purified oligonucleotide sample.

### 2.5. Preparation of DNA Structures

DNA structures were prepared via annealing of each fluorescent and/or 5′-^32^P-labeled oligonucleotide with a complementary one according to desired composition of a DNA oligonucleotide (see Table 1) [20]. The oligonucleotides were incubated for 5 min at 95 °C and cooled slowly to room temperature. The degree of hybridization was monitored by electrophoresis in a 10% polyacrylamide gel (acrylamide/bis-acrylamide = 40:1) at 4 °C in TBE buffer (89 mM Tris, 89 mM H_3_BO_3_, 2 mM EDTA, pH 8.3). All DNA structures were obtained with a high yield and were stable under these conditions.

### 2.6. Endonuclease Assay

FEN1 nuclease activity is highly sensitive to salt concentration (see Figure S1), and therefore KCl concentration in all reaction mixtures was adjusted to 100 mM. For this purpose, we mixed reactions with 50 mM KCl and then added protein dilution buffer (buffer A with 0.6 mg/mL BSA) in an amount required to obtain final concentration 100 mM KCl taking into account salt from protein aliquots. Thus, reaction mixtures (20 µL) composed of buffer A, at least 0.6 mg/mL BSA (BSA amount increased due to added volume of protein dilution buffer), 5 mM MgCl_2_, 10 nM DNA, and 10 nM FEN1 were equilibrated for 5 min on ice and then were incubated at 37 °C for the periods indicated in figure legends (2, 5, 7, 10, 15, or 30 min). In the case of experiments on an XPA influence, reaction mixtures containing 8 nM FEN1 and 10, 40, 100, or 300 nM XPA were also equilibrated for 5 min on ice and then were incubated at 37 °C for 7 min. The reactions were terminated with 8 µL of hot formamide (95 °C), and the mixtures were heated to 95 °C until volume decreased via evaporation to 10 µL. Then, the reaction mixtures were loaded onto denaturing 10% polyacrylamide gel (acrylamide/bis-acrylamide = 19:1, 8 M urea), and separated by electrophoresis in TBE buffer. The cleavage products were detected in the FAM mode on a Typhoon FLA 9500 (GE Healthcare) and quantified using Quantity One software (v. 4.6.7., Bio-Rad).

### 2.7. Electrophoretic Mobility Shift Assay (EMSA)

Reaction mixtures (10 µL) consisting of buffer A, 0.6 mg/mL BSA, 10 nM DNA, 0.1 nM pUC19 plasmid (as a competitor DNA), 5 mM MgCl_2_ (only for XPA-binding experiments), 5 mM CaCl_2_ (where indicated), and proteins at the indicated concentrations were incubated for 20 min at 37 °C. Experiments with Flu-XPA were performed without DNA in buffer A with 0.6 mg/mL BSA. Then, 2 µL of loading buffer (20% of glycerol, 0.015% of bromophenol blue in buffer A with 0.6 mg/mL BSA) at 37 °C was added, and the samples were immediately put on ice. Next, the samples were loaded onto a 5% native polyacrylamide gel (7% for Flu-XPA-containing samples; acrylamide/bis-acrylamide = 60:1) and separated by electrophoresis in TBE at 4 °C with a voltage decrease of 10–12 V/cm. In the case of fluorescein-containing DNA or Flu-XPA, gels were scanned between glass plates in the FAM mode on the Typhoon FLA 9500 (GE Healthcare). The gels with 5′-^32^P-labeled DNA were dried, and a phosphor screen was exposed to them overnight. Electrophoretic bands were quantified using the Typhoon FLA 9500 (GE Healthcare) and Quantity One software.

### 2.8. Far-Western Blotting Assay (Dot Blot Protein–Protein Binding Assay)

The increased amounts (1, 3, or 5 µL) of proteins (XPA 1.4 µM, RPA 5 µM, FEN1 5 µM, and BSA 5 µM) were dotted on a dry nitrocellulose membrane. For each target experimental membrane, a membrane of an antibody cross-reactivity control was set up. The membranes were then blocked in blocking buffer (5% milk in TBST) for 90 min at 4 °C and washed 3 times with TBS for 5 min each. After that, target membrane was incubated with 0.3 µM XPA in a buffer (50 mM Tris-HCl 7.5, 100 mM NaCl, 1 mM DTT, and 0.6 g/L BSA) and washed in the same way. Both membranes’ were incubated with a primary antibody (anti-XPA, mouse Ab-1, 5A2, Calbiochem, San Diego, CA, USA) in TBS overnight at 4 °C and subsequently washed in the same manner. Then, the membranes were incubated (for 1 h at 4 °C) with a secondary antibody conjugated with horseradish peroxidase and also washed 3 times with TBS for 5 min each. Visual detection was carried out using the Pico Plus Reagent Kit (Thermo Scientific, Waltham, MA, USA) and the gel documentation system (Amersham Imager 600, GE Healthcare).

### 2.9. Statistical Analysis

In all experiments, amounts of a substrate and product were quantified, and the percentage of substrate cleavage was determined as the product/(substrate + product) ratio. This method allowed for correcting data for any loading error among lanes. Moreover, data from the experiments on the XPA and RPA influence were normalized to the percentage of FEN1 cleavage product without a second protein. All assays were performed at least in triplicate, and representative gels are shown. The data were quantified in Quantity One software and analyzed in Microsoft Excel. EMSA and cleavage data are displayed as means ± SD. The *p*-values were determined at nearly 50% of product or complex formation using an unpaired two-tailed *t*-test.

## 3. Results

### 3.1. FEN1 Is Able to Cleave Flap Substrates Containing a Bulky Lesion in Combination with a Gap of Different Sizes at Nearly Physiological Salt Conditions

The FEN1 crystal structure [44] reveals that the interaction with a duplex part of a DNA substrate is mediated by K^+^ ions and facilitates a scissile bond’s positioning in the active site. Indeed, K^+^ ions are the most abundant cations in the intracellular fluid [45] with a physiological concentration of 140–150 mM [46]. On the other hand, low-salt conditions (50 mM and less) are commonly used for FEN1 nuclease assays (e.g., [47]). Accordingly, we started our experiments by investigating the contribution of salt concentration to FEN1’s binding affinity and cleavage efficiency (Figure S1A). As expected, these experiments showed that FEN1 catalytic activity is much lower at K^+^ concentrations of 100–120 mM compared to 20 and 50 mM. The binding affinity decreasing was substantial only between salt concentrations 20 and 50 mM (Figure S1B). DNA binding by FEN1 at K^+^ concentrations higher than 50 mM is nearly equal, but catalytic efficiency was different. According to an existing model of FEN1 catalytic reaction, FEN1 binds primarily to duplex bases of the flap, bends the substrate with the 5′ single-stranded flap being directed under the cap domain, while two base pairs unpairing promotes correct positioning of the scissile bond [44]. Our results indicated that stages after initial duplex binding are strongly K^+^-sensitive, possibly because this ion stabilizes duplex structure and prevents its bending and base pairs unpairing. All subsequent experiments were performed with reaction mixtures normalized to 100 mM K^+^. This concentration was chosen from the standpoint of a balance between convenient FEN1 catalytic activity detection and physiological K^+^ concentration.

Previous studies have demonstrated that FEN1 can cleave long flap substrates containing a single *cis*-diamminedichloroplatinum (CDDP) adduct or a branched flap without additional modifications [48]. By contrast, two CDDP adducts within the flap or CDDP-adducted branched flaps inhibit FEN1 activity [48], similarly to a flap substrate blocked by streptavidin [49]. Basically, any flap modification that leads to the flap’s inability to be bent by FEN1 results in cleavage inhibition. In our study, DNA structures contain fluorescein on a flexible linker (Table 1); hence, we assumed that this lesion does not dramatically change flap structure’s flexibility. Indeed, fluorescein substitution inside the flap does not affect FEN1 cleavage efficiency and binding affinity (Figure S2). These results motivate us to use DNAs bearing fluorescein substitution inside a 5′-flap as a bulky lesion and a reporter group simultaneously.

**Figure 1:**
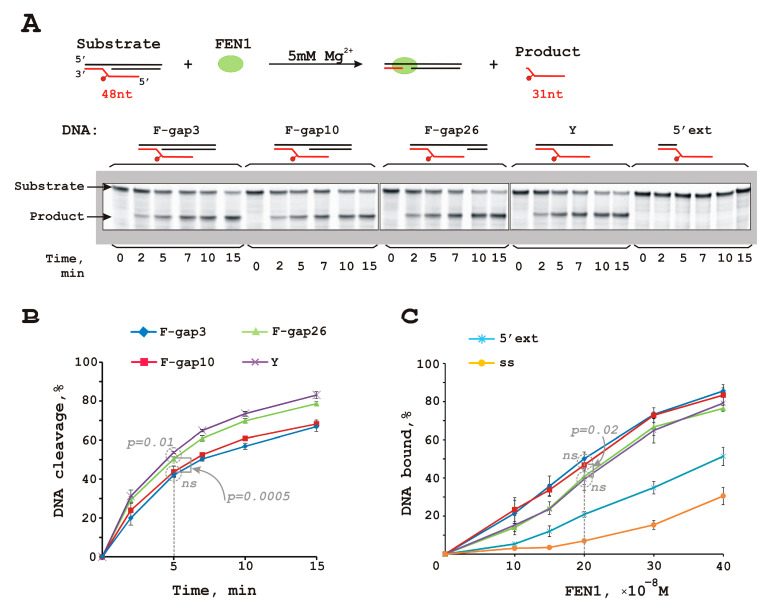
FEN1 cleaves DNAs containing a damaged flap and gap. FEN1 can cleave a flap bearing a fluorescein-substituted dUMP analog in combination with a gap (**A**,**B**). The gap size does not affect FEN1’s binding affinity (**C**), but the presence of both template strand parts forming the flap basis is indispensable for the correct FEN1–DNA interaction. Gels for (**C**) panel are presented at Figure S2B. The ssDNA was an oligonucleotide Fg (see Table S1) used for the 5′-flap structure generation. *p*-values marked as *p* on the plots (**A**,**B**); ns means not sufficient. Reaction mixtures (20 µL) contained FEN1 (10 nM), a DNA substrate (10 nM), 5 mM Mg^2+^ (for nuclease experiments), 50 mM Tris-HCl pH 7.5, 1 mM dithiothreitol, at least 0.6 mg/mL BSA, and 100 mM KCl, and the reactions were performed as described in the Section 2—Materials and Methods Section. Substrate and cleavage product sizes are indicated. Schematic representation of the DNA substrates is depicted above the figure. Raw data can be viewed in Supplementary Materials.

Further, we examined FEN1 ability to catalyze flap cleavage regardless of gap size: 3, 10, or 26 nt or Y-shaped structure (Figure 1).

Cleavage efficiency unexpectedly increased with increasing gap size: F-gap3 = F-gap10 < F-gap26 < Y-shape. This is surprising because according to FEN1’s optimal substrate requirements, the substrate should contain a short 5′-flap with a one-nucleotide 3′-flap without a gap (this substrate mimics a replication intermediate) [50,51]; the F-gap3 structure is the best match for this model. At the same time, FEN1’s binding affinity order for these DNA structures is F-gap26 = Y-shape < F-gap3 = F-gap10 (Figure 1C and Figure S3). Thus, dsDNA containing two-way junctions (with 5′ and 3′ single-strand extensions) is the key structural element for FEN1’s ability to bind DNA. This observation is in agreement with existing biochemical data [49] and FEN1 crystal structure [44].

DNA structure without an upstream template strand (5′ext) was bound by FEN1 with lower affinity and was not digested (Figure 1 and Figure S3). Consequently, the presence of both template strand parts forming a flap basis was indispensable for the formation of a cleavage-competent complex. The structural features of DNA substrates containing a single-stranded flap exposed from the template and duplex sharp bending with dsDNA segments on both sides of the flap provided FEN1 substrate specificity [44]. Nevertheless, there are discrepancies among reports about FEN1’s ability to cleave 5′-extentions (can cleave, [52], and cannot cleave, [53]) and Y-shape structures (can cleave, [52], and cannot cleave, [53]).

Mg^2+^-dependent conformational changes in DNA are indispensable for the formation of a final catalytically active complex [47,54]. Assuming that the complex of a DNA substrate with FEN1-Ca^2+^ better adopts the catalytically viable conformation as compared to the one without a divalent metal, we also performed DNA-binding experiments in a Ca^2+^-containing buffer (Figure S3B). Under these conditions, FEN1 bound to DNAs with higher affinity (Figure S3D) but retained the same specificity (Figure S3E). Notably, the nuclease activity of FEN1 is functional only in the presence of Mg^2+^ and Mn^2+^ ions and is not supported by Ca^2+^ [53]. Moreover, Ca^2+^ ions inhibited the reaction even when Mg^2+^ and Mn^2+^ ions were present (Figure S4). In vivo, FEN1 avoids inhibition by calcium owing to low intracellular concentration of Ca^2+^: 0.1 µM [55].

### 3.2. FEN1 Interacts with XPA in DNA-Independent Manner

**Figure 2:**
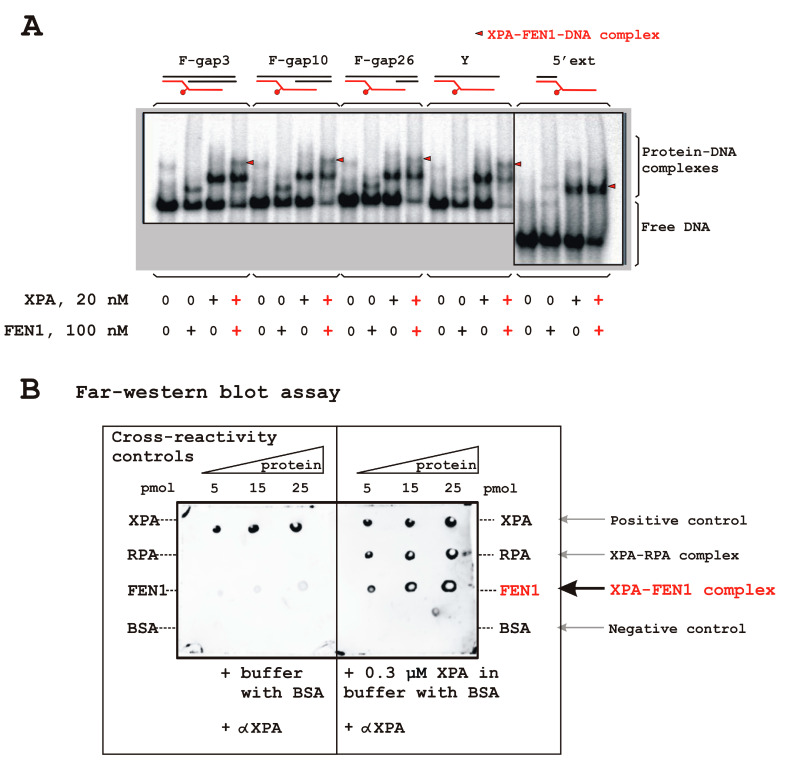
XPA–FEN1 interaction. An XPA–FEN1–DNA complex observed for DNAs with the different gap sizes (**A**). Schematic representation of the DNA substrates is given above the figure. XPA and FEN1 interact directly in the absence of DNA (**B**). For details, see Section 2. αXPA: an anti-XPA antibody. Raw data can be viewed in Supplementary Materials.

Next, we tested whether XPA and/or RPA interacts with FEN1 during DNA binding. Using all the DNA structures, by EMSA, we registered a band with lower mobility as compared to the FEN1–DNA or XPA–DNA complex; this band was assumed to be the XPA–FEN1–DNA ternary complex (Figure 2A and Figure S5A, in Ca^2+^ presence). Note, the FEN1–XPA–DNA complex was formed equally with damaged and undamaged flap structures (Figure S5B) and XPA does not discriminate damage inside flap (Figure S5C). The lower-mobility band was also observed with bubbled DNA (Figure S5D). Two possible scenarios for the ternary complex formation could be proposed: either both proteins bind to the same DNA molecule independently, or some protein–protein interactions take place inside the complex (each protein binds to the DNA and interacts with the other protein simultaneously; one protein binds to the DNA and the second protein enters the complex via protein–protein interactions). To examine whether XPA and FEN1 contact directly, we performed a far-Western blotting assay (Figure 2B). Various amounts of proteins were applied to a nitrocellulose membrane, which was incubated ether with 0.3 µM XPA (right panel) or with the buffer without XPA (left panel) followed by treatment with anti-XPA antibody (see Materials and Methods, Section 2.8). XPA and BSA were used as positive and negative controls, respectively. These experiments confirmed XPA–FEN1 complex formation in the absence of DNA.

Previously, RPA was shown to compete with FEN1 for the binding to a long flap 1 [37]. In our study, we examine FEN1 and RPA binding to flap and/or gap ssDNA platforms in one DNA molecule (Figure S6). Regardless of numbers and locations of binding platforms (an ssDNA platform inside the gap and flap itself), RPA competed with FEN1 for binding and displaced it from DNA at increasing RPA concentrations. Of note, RPA prefers a gap platform to bind [20], and this binding position also inhibits DNA cleavage by FEN1, especially in the case of the F-gap26 structure (Figure S6B).

### 3.3. RPA–XPA–FEN1 Complex Formation

**Figure 3:**
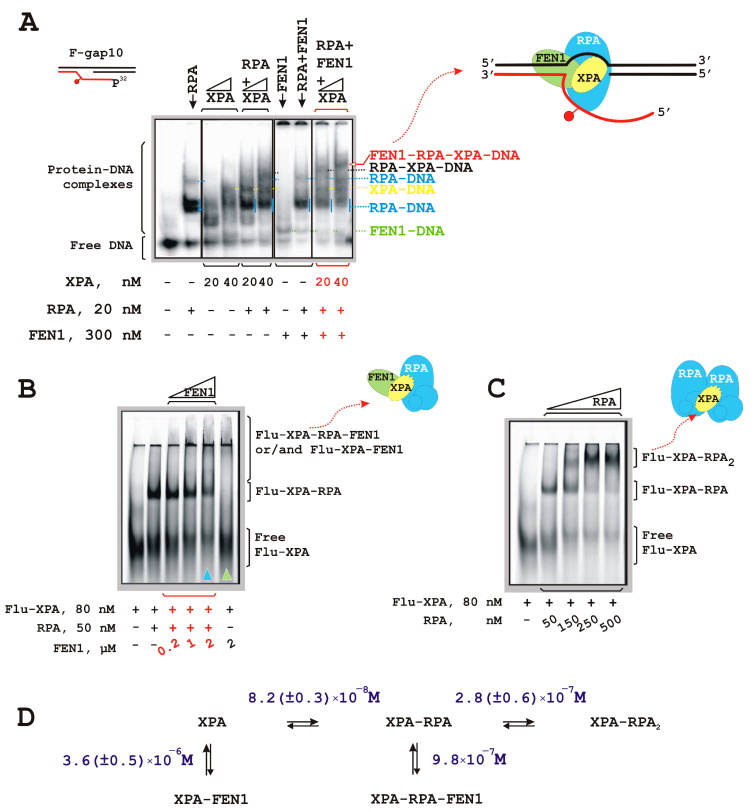
XPA interacts with both RPA and FEN1. XPA is able to recruit FEN1 and RPA to DNA simultaneously to arrange an XPA–FEN1–RPA–DNA complex (**A**). Fluorescently labeled XPA can arrange the Flu-XPA–FEN1–RPA complex (**B**). Free Flu-XPA disappearance is more pronounced under conditions of simultaneous presence of FEN1, XPA, and RPA in the reaction mixture (this lane is designated by blue triangle) compared to the lane containing just Flu-XPA+FEN1 (green triangle). The XPA is able to bind two RPA molecules simultaneously (**C**). Representation of putative XPA–FEN1–RPA–DNA, XPA–FEN1–RPA, and XPA–RPA_2_ complexes are depicted. (**D**) EC_50_ values for complexes of Flu-XPA are shown. For details, please see Section 2. Raw data can be viewed in Supplementary Materials.

Under conditions of simultaneous XPA, FEN1, and RPA presence in the reaction mixture, a putative XPA–FEN1–RPA–DNA complex was detectable (Figure 3A). This complex does not correspond to these proteins’ ternary complexes, which allows for us to propose that it is quaternary with some degree of confidence. We took into consideration that this complex may be organized by XPA because we failed to detect any RPA contact with FEN1.

To further analyze the possibility of XPA–FEN1–RPA complex formation, we used a fluorescently labeled XPA protein (Flu-XPA). Flu-XPA molecules entered a native gel in the absence of DNA (Figure 3B). In the presence of RPA, an additional band was observed corresponding to a Flu-XPA–RPA complex with 1:1 stoichiometry. Unfortunately, subsequent Flu-XPA–RPA complex titration with increasing concentrations of FEN1 did not result in XPA–FEN1–RPA complex formation. Instead, the titration resulted in disappearance of Flu-XPA and Flu-XPA–RPA complexes and accumulation of some complexes in the loading wells. Titration of Flu-XPA by FEN1 also yielded bands at the top of the gel. We suggest that complexes with FEN1 cannot enter the gel due to their insolubility under the EMSA conditions (pI value of FEN1 8.8 is near pH value in the electrophoretic buffer at 4 °C).

We examined the disappearance of free Flu-XPA and found that it was more pronounced in the lane containing Flu-XPA, RPA, and 200 nM FEN1 compared to the lane with Flu-XPA and 200 nM FEN1 only. Thus, the XPA–FEN1 interaction becomes more effective in RPA presence. Notably, the band corresponding to the XPA–FEN1–RPA–DNA complex was also clearly detectable.

Overall, we assumed the existence of an XPA–FEN1–RPA complex where XPA accommodates FEN1 and RPA contacts simultaneously. RPA can stabilize this complex, and the XPA–RPA stabilization is in agreement with XPA’s scaffolding function inside NER.

To further clarify the XPA ability to interact with two other protein molecules simultaneously, we performed Flu-XPA titration with RPA (Figure 3C). These experiments revealed that one XPA molecule is able to interact with two RPA molecules simultaneously. At equimolar amounts of Flu-XPA and RPA, a bimolecular Flu-XPA–RPA complex was detected. Subsequent titration led to Flu-XPA–RPA_2_ complex formation at a high RPA excess. Half-maximal effective concentration (EC_50_) for the binding of the first RPA molecule was roughly three times lower compared with EC_50_ for the second RPA molecule to join (Figure 3D). Taking into account that each RPA-binding motif on XPA is occupied by a single separate RPA molecule, we propose that EC_50_ for the second RPA molecule reflects an interaction of XPA’s DNA-binding domain (XPA-DBD) with RPA70AB [27]. Physical interaction between the N terminus of XPA (XPA-N) and RPA32C is stronger than the XPA-DBD–RPA70AB contact and is important for the initial association. On the other hand, subsequent XPA-DBD association with RPA70AB is needed for structural organization of the NER complex. The binding of the first RPA molecule may involve simultaneous contacts of XPA-N with RPA32C and XPA-DBD with RPA70AB. In the case of FEN1 joining the Flu-XPA–RPA complex, EC_50_ was approximately four times lower compared to the bimolecular association between FEN1 and Flu-XPA. These data are also consistent with a stronger XPA–FEN1 interaction in the presence of RPA.

### 3.4. XPA Moderately Inhibits FEN1 Catalytic Activity

**Figure 4:**
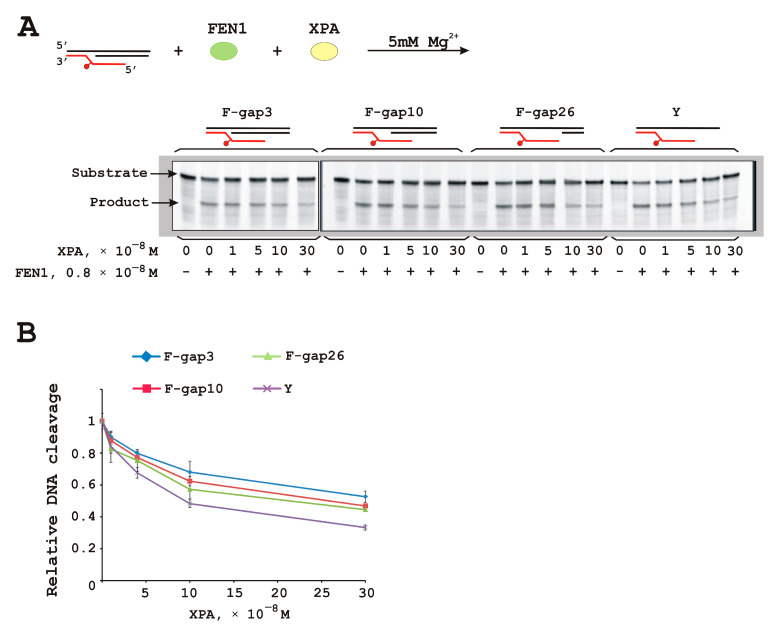
XPA inhibits FEN1 activity on all DNA substrates. The endonuclease activity of FEN1 was assessed on DNAs with different gap sizes in the presence of different amounts of XPA (**A**). Schematic representation of the DNA substrates is given above the figure. A twofold decrease in reaction products was observed in the presence of a large XPA molar excess (**B**). Results of the XPA influence were normalized to the percentage of FEN1 cleavage product without XPA. Reaction mixtures (20 µL) contained FEN1 (8 nM), a DNA substrate (10 nM), an indicated amount of XPA, 5 mM Mg^2+^, 50 mM Tris-HCl 7.5, 1 mM dithiothreitol, at least 0.6 mg/mL BSA, and 100 mM KCl, and the reactions were performed as described in Section 2. Raw data can be viewed in Supplementary Materials.

Following the idea that the XPA–FEN1 interaction may exist at the resynthesis stage of NER, we tested whether XPA affects FEN1 nuclease activity on gap- and flap-containing DNAs. We use conditions where FEN1 digested approximately half of a substrate for subsequent XPA titration experiments (Figure 4). Of note, only a twofold decrease in the yield of the reaction product was observed at a large XPA molar excess (more than tenfold over FEN1). The inhibitory effect was greater with DNA containing long gaps (Figure 4B). The number or size of the reaction products did not change, and therefore FEN1 specificity remained the same. We propose that the observed inhibitory effect is explained by XPA competing both with DNA for FEN1 binding and with FEN1 for DNA binding.

At the same time, RPA’s inhibitory effect could be interpreted as simple displacement by the DNA-binding protein. Under conditions of simultaneous presence of RPA and XPA in the reaction mixture, the inhibition profile matched the one for RPA alone (Figure S7).

## 4. Discussion

XPA is one of the smallest proteins of the NER machine, but it is very important for the process functioning. Mutations in the *XPA* gene lead to the most severe forms of xeroderma pigmentosum [17,56]. For a long time, the only function of XPA was considered to be participation in the NER process, and initially it was assigned the role of a damage-recognizing factor [57,58]. As data accumulated, this hypothesis was replaced by the assumption that XPA acts as a hinge that ensures interaction between other participants in the process [59]. In addition to the proteins of the NER complex, XPA has been found to interact with the cell cycle control proteins (reviewed in [19]), as well as with the replication factor PCNA [23,24,25]. Thus, XPA can be considered not only as the NER factor, but also as a component of the replication machinery and probably other cellular processes.

Here, we demonstrated an interaction of XPA with FEN1 both in the presence and absence of DNA (Figure 2). FEN1 activity is important for several DNA metabolic processes [10,60,61]. The structure-specific mechanism of flap cleavage allows for FEN1 to cleave a 5′-flap on a variety of DNA replication and repair intermediates. During replication, FEN1 processes intermediates of Okazaki fragment 5′-flaps thereby generating nicked DNA products that can be ligated for Okazaki fragment maturation. FEN1 also operates in long-patch BER, sequence duplication prevention, trinucleotide repeat expansion limiting [13,62,63,64,65], and during telomere maintenance [66,67,68,69]. The involvement of FEN1 in so many distinct cellular pathways is regulated by different protein partners that dictate FEN1’s cellular location, its recruitment into protein ensembles, and its structure tuning by post-translational modifications and affect its nuclease activity [60].

Structural organization of the XPA–FEN1–DNA complex is not obvious. We propose the spatial organization of the complex on the model DNA used in this study from the standpoint that both proteins are bound to DNA (Figure S8). FEN1 binds to the flap basis, forming contacts with the duplex part and the template ssDNA part inside the gap. Thus, XPA is forced to bind to the opposite ss-ds DNA junction with a 3′-ssDNA gap extension. Note that XPA binds specifically to the ss-ds DNA junction with a 5′-extension where XPA can stabilize the interaction by intercalation of a Trp residue between unpaired bases of the single-stranded extension [70,71].

One of the main partners of XPA in the NER process is RPA. The XPA–RPA complex as well as a ternary complex with DNA were shown earlier [72,73], and XPA interaction with RPA is indispensable for NER reaction [26,27]. XPA interacts with two of three RPA subunits: RPA70 by the globular core XPA domain and RPA32. It was recently demonstrated that the interaction with RPA32 subunit may be important for the initial step of NER complex formation, whereas the interaction with RPA70 is needed for structural shaping of the complex [27]. It is reported that an XPA dimer can form a complex with one RPA molecule [74]. Does each XPA molecule come into contact with each RPA molecule in this complex type or only with a single one? Our experiments revealed a reverse situation: one XPA molecule is able to interact with two RPA molecules simultaneously (Figure 3C). Because this complex was registered during the titration of FAM–XPA with RPA, we suggest that both RPA molecules interact with one XPA molecule through subunit RPA70 of one of the RPAs and subunit RPA32 of the other one. We also cannot rule out the possibility that in the XPA–RPA_2_ complex, two RPA molecules interact with each other. Interactions between RPA molecules were recently described [75]. In the case of the XPA–FEN1–RPA complex, XPA may interact with RPA70 subunit via globular domain and with FEN1 via N-terminus. Overall, these data illustrate XPA’s function as linchpin protein, which accommodates two (or more) proteins simultaneously within protein ensemble. Furthermore, our findings indicate that the RPA–XPA interaction may stabilize contacts of XPA (and possibly RPA) with other proteins, so serving as a platform to arrange multi-protein complexes.

**Figure 5:**
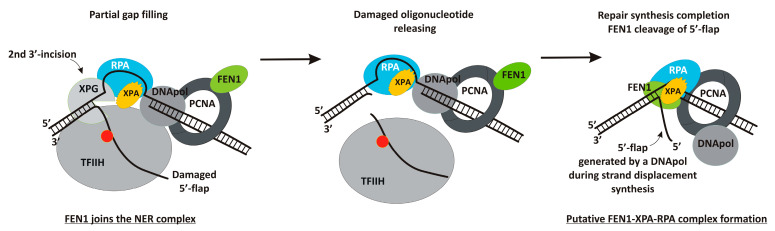
Putative model of XPA involvement in the post-incision NER stages. The first incision by XPF-ERCC1 allows for the recruitment of the resynthesis factors. FEN1 joins the NER complex together with replication machine. The repair synthesis can be initiated and proceed halfway through the gap in the absence of the second XPG incision [1]. XPA can remain in the NER complex via a protein–protein interaction with RPA [20]. Following second excision by XPG, the lesion-containing oligonucleotide is released with TFIIH bound to it. The ongoing Polδ synthesis could originate a “nick translation” and FEN1 works in association with the Polδ and Ligase I to limit this process and allow for ligation [7,36]. We suggest that the XPA–FEN1–RPA complex can be formed at these stages. Our DNA structures could model both intermediates containing a 5′-flap: before XPG incision and after strand displacement.

Notably, RPA also organizes the XPA–RPA–XPG complex [28]. Although XPG and XPA are present together in several protein complexes at different NER stages, they do not interact directly [28]. XPA’s location close to the PCNA recruitment point allows for proposing that XPA recruits PCNA into the NER complex. Thereafter, FEN1 could arrive as a PCNA partner [36], and subsequently, the XPA–FEN1–RPA complex can be formed. Based on XPA and FEN1 physical and functional interactions as well as ternary XPA–FEN1–RPA complex formation, a putative model of XPA interaction with protein partners in the post-incision complexes of the NER process can be supposed (Figure 5).

The scaffolding function of XPA is absolutely indispensable for both NER sub-pathways: global genome NER and transcription-coupled NER (TC-NER). The TC-NER rapidly eliminates transcription-blocking lesions from actively transcribed DNA strands [56,76,77]. Transcription stalling at a site of a lesion can give rise to R-loops too [15,78]. Thus, protein factors of TC-NER machinery can be easily recruited to the R-loop site. A recent paper showed that FEN1 can participate in R-loop resolution [13,16]. Structure-specific NER endonuclease XPF–ERCC1 is also involved in R-loop cleavage [16]. Because XPA interacts with both proteins, it is possible to assume that XPA can be involved in the R-loop resolving complex in the same way as it functions in the NER complex [79]. 

Altogether, our current results are consistent with an idea of XPA involvement in processes where FEN1 is functionally active. Further experiments in this field may validate this assumption.

## Figures and Tables

**Table 1:** Structures of Model DNA Used in the Study.

DNA Designation	Oligo Composition	Schematic View	Gap Size
**F-gap3**	60 Fg 40	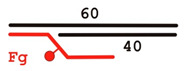	3
**F-gap10**	60 Fg 33	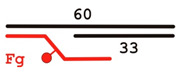	10
**F-gap26**	60 Fg 17	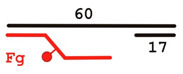	26
**Y**	60 Fg	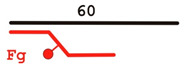	−
**5’ext**	17up Fg	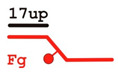	−
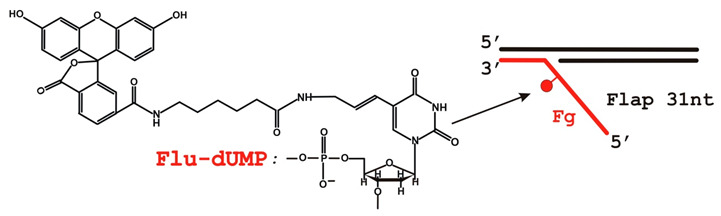			

## Data Availability

All data necessary to reproduce our results are included in this published article (and its Supplementary Information File). Raw data are available.

## Supplementary Materials

The following supporting information can be downloaded at: https://www.mdpi.com/article/10.3390/biom14070814/s1, Figure S1: The monovalent ions inhibit FEN1-catalyzed DNA hydrolysis; Figure S2: The fluorescein substitution does not affect FEN1 cleavage efficiency and binding affinity; Figure S3: The gap size does not affect FEN1 binding affinity, but the presence of both template strand parts forming flap basis are indispensable for correct FEN1-DNA interaction; Figure S4: The divalent metal ions affect differently on FEN1 nuclease activity; Figure S5: FEN1-XPA-DNA complex is formed equally with different sizes gap DNAs in the presence of calcium ions; Figure S6: RPA competes with FEN1 for binding and displace it from DNA (FEN1-DNA complex titration by the increasing amount of RPA was resulted in FEN1 complex disappearance); Figure S7: Inhibition of FEN1 catalytic activity by RPA is classical for displacement by DNA binding protein; Figure S8: Spatial organization of the putative FEN1–XPA–DNA complex on the flapped DNA; Table S1: Oligonucleotide sequences. Original Western Blots images.

## References

[1] Staresincic L., Fagbemi A.F., Enzlin J.H., Gourdin A.M., Wijgers N., Dunand-Sauthier I., Giglia-Mari G., Clarkson S.G., Vermeulen W., Schärer O.D. (2009). Coordination of dual incision and repair synthesis in human nucleotide excision repair. EMBO J..

[2] Schärer O.D. (2013). Nucleotide excision repair in eukaryotes. Cold Spring Harb. Perspect. Biol..

[3] Fagbemi A.F., Orelli B., Schärer O.D. (2011). Regulation of endonuclease activity in human nucleotide excision repair. DNA Repair.

[4] Ogi T., Limsirichaikul S., Overmeer R.M., Volker M., Takenaka K., Cloney R., Nakazawa Y., Niimi A., Miki Y., Jaspers N.G. (2010). Three DNA polymerases, recruited by different mechanisms, carry out NER repair synthesis in human cells. Mol. Cell.

[5] Sakurai S., Kitano K., Yamaguchi H., Hamada K., Okada K., Fukuda K., Uchida M., Ohtsuka E., Morioka H., Hakoshima T. (2005). Structural basis for recruitment of human flap endonuclease 1 to PCNA. EMBO J..

[6] Querol-Audí J., Yan C., Xu X., Tsutakawa S.E., Tsai M.S., Tainer J.A., Cooper P.K., Nogales E., Ivanov I. (2012). Repair complexes of FEN1 endonuclease, DNA, and Rad9-Hus1-Rad1 are distinguished from their PCNA counterparts by functionally important stability. Proc. Natl. Acad. Sci. USA.

[7] Lancey C., Tehseen M., Raducanu V.S., Rashid F., Merino N., Ragan T.J., Savva C.G., Zaher M.S., Shirbini A., Blanco F.J. (2020). Structure of the processive human Pol δ holoenzyme. Nat. Commun..

[8] Blair K., Tehseen M., Raducanu V.S., Shahid T., Lancey C., Rashid F., Crehuet R., Hamdan S.M., De Biasio A. (2022). Mechanism of human Lig1 regulation by PCNA in Okazaki fragment sealing. Nat. Commun..

[9] Tsutakawa S.E., Tainer J.A. (2012). Double strand binding-single strand incision mechanism for human flap endonuclease: Implications for the superfamily. Mech. Ageing Dev..

[10] Balakrishnan L., Bambara R.A. (2013). Flap endonuclease 1. Annu. Rev. Biochem..

[11] Fortini P., Dogliotti E. (2007). Base damage and singlestrand break repair: Mechanisms and functional significance of short- and long-patch repair subpathways. DNA Repair.

[12] Krokan H.E., Bjørås M. (2013). Base excision repair. Cold Spring Harb. Perspect. Biol..

[13] Laverde E.E., Lai Y., Leng F., Balakrishnan L., Freudenreich C.H., Liu Y. (2020). R-loops promote trinucleotide repeat deletion through DNA base excision repair enzymatic activities. J. Biol. Chem..

[14] Laverde E.E., Polyzos A.A., Tsegay P.P., Shaver M., Hutcheson J.D., Balakrishnan L., McMurray C.T., Liu Y. (2022). Flap Endonuclease 1 Endonucleolytically Processes RNA to Resolve R-Loops through DNA Base Excision Repair. Genes.

[15] Garcia-Muse T., Aguilera A. (2019). R Loops: From Physiological to Pathological Roles. Cell.

[16] Cristini A., Ricci G., Britton S., Salimbeni S., Huang S.N., Marinello J., Calsou P., Pommier Y., Favre G., Capranico G. (2019). Dual Processing of R-Loops and Topoisomerase I Induces Transcription-Dependent DNA Double-Strand Breaks. Cell Rep..

[17] Sugitani N., Sivley R.M., Perry K.E., Capra J.A., Chazin W.J. (2016). XPA: A key scaffold for human nucleotide excision repair. DNA Repair.

[18] Borszéková Pulzová L., Ward T.A., Chovanec M. (2020). XPA: DNA Repair Protein of Significant Clinical Importance. Int. J. Mol. Sci..

[19] Krasikova Y.S., Lavrik O.I., Rechkunova N.I. (2022). The XPA Protein-Life under Precise Control. Cells.

[20] Krasikova Y.S., Rechkunova N.I., Maltseva E.A., Lavrik O.I. (2018). RPA and XPA interaction with DNA structures mimicking intermediates of the late stages in nucleotide excision repair. PLoS ONE.

[21] Kim H.S., Brill S.J. (2001). Rfc4 interacts with Rpa1 and is required for both DNA replication and DNA damage checkpoints in Saccharomyces cerevisiae. Mol. Cell Biol..

[22] Hayner J.N., Douma L.G., Bloom L.B. (2014). The interplay of primer-template DNA phosphorylation status and single-stranded DNA binding proteins in directing clamp loaders to the appropriate polarity of DNA. Nucleic Acids Res..

[23] Gilljam K.M., Feyzi E., Aas P.A., Sousa M.M., Muller R., Vagbo C.B., Catterall T.C., Liabakk N.B., Slupphaug G., Drablos F. (2009). Identification of a novel, widespread, and functionally important PCNA-binding motif. J. Cell Biol..

[24] Gilljam K.M., Müller R., Liabakk N.B., Otterlei M. (2012). Nucleotide excision repair is associated with the replisome and its efficiency depends on a direct interaction between XPA and PCNA. PLoS ONE.

[25] Hara K., Uchida M., Tagata R., Yokoyama H., Ishikawa Y., Hishiki A., Hashimoto H. (2018). Structure of proliferating cell nuclear antigen (PCNA) bound to an APIM peptide reveals the universality of PCNA interaction. Acta Cryst. F Struct. Biol. Commun..

[26] Topolska-Woś A.M., Sugitani N., Cordoba J.J., Le Meur K.V., Le Meur R.A., Kim H.S., Yeo J.E., Rosenberg D., Hammel M., Schärer O.D. (2020). A key interaction with RPA orients XPA in NER complexes. Nucleic Acids Res..

[27] Kim M., Kim H.S., D′Souza A., Gallagher K., Jeong E., Topolska-Wós A., Ogorodnik Le Meur K., Tsai C.L., Tsai M.S., Kee M. (2022). Two interaction surfaces between XPA and RPA organize the preincision complex in nucleotide excision repair. Proc. Natl. Acad. Sci. USA.

[28] He Z., Henricksen L.A., Wold M.S., Ingles C.J. (1995). RPA involvement in the damage-recognition and incision steps of nucleotide excision repair. Nature.

[29] Li L., Elledge S.J., Peterson C.A., Bales E.S., Legerski R.J. (1994). Specific association between the human DNA repair proteins XPA and ERCC1. Proc. Natl. Acad. Sci. USA.

[30] Croteau D.L., Peng Y., Van Houten B. (2008). DNA repair gets physical: Mapping an XPA-binding site on ERCC1. DNA Repair.

[31] de Laat W.L., Appeldoorn E., Sugasawa K., Weterings E., Jaspers N.G., Hoeijmakers J.H. (1998). DNA-binding polarity of human replication protein A positions nucleases in nucleotide excision repair. Genes. Dev..

[32] Dunand-Sauthier I., Hohl M., Thorel F., Jaquier-Gubler P., Clarkson S.G., Scharer O.D. (2005). The spacer region of XPG mediates recruitment to nucleotide excision repair complexes and determines substrate specificity. J. Biol. Chem..

[33] Tsodikov O.V., Ivanov D., Orelli B., Staresincic L., Shoshani I., Oberman R., Schärer O.D., Wagner G., Ellenberger T. (2007). Structural basis for the recruitment of ERCC1-XPF to nucleotide excision repair complexes by XPA. EMBO J..

[34] Krasikova Y.S., Rechkunova N.I., Maltseva E.A., Petruseva I.O., Lavrik O.I. (2010). Localization of xeroderma pigmentosum group A protein and replication protein A on damaged DNA in nucleotide excision repair. Nucleic Acids Res..

[35] Fadda E. (2013). Conformational determinants for the recruitment of ERCC1 by XPA in the nucleotide excision repair (NER) Pathway: Structure and dynamics of the XPA binding motif. Biophys. J..

[36] Mocquet V., Lainé J.P., Riedl T., Yajin Z., Lee M.Y., Egly J.M. (2008). Sequential recruitment of the repair factors during NER: The role of XPG in initiating the resynthesis step. EMBO J..

[37] Nazarkina J.K., Petrousseva I.O., Safronov I.V., Lavrik O.I., Khodyreva S.N. (2003). Interaction of flap endonuclease-1 and replication protein A with photoreactive intermediates of DNA repair. Biochemistry.

[38] Laemmli U.K. (1970). Cleavage of structural proteins during the assembly of the head of bacteriophage T4. Nature.

[39] Krasikova Y.S., Rechkunova N.I., Maltseva E.A., Petruseva I.O., Silnikov V.N., Zatsepin T.S., Oretskaya T.S., Schärer O.D., Lavrik O.I. (2008). Interaction of nucleotide excision repair factors XPC-HR23B, XPA, and RPA with damaged DNA. Biochemistry.

[40] Maltseva E.A., Krasikova Y.S., Naegeli H., Lavrik O.I., Rechkunova N.I. (2014). Effect of point substitutions within the minimal DNA-binding domain of xeroderma pigmentosum group A protein on interaction with DNA intermediates of nucleotide excision repair. Biochemistry.

[41] Henricksen L.A., Umbricht C.B., Wold M.S. (1994). Recombinant replication protein A: Expression, complex formation, and functional characterization. J. Biol. Chem..

[42] Moor N.A., Vasil′eva I.A., Anarbaev R.O., Antson A.A., Lavrik O.I. (2015). Quantitative characterization of protein-protein complexes involved in base excision DNA repair. Nucleic Acids Res..

[43] Sambrook J., Fritsch E.F., Maniatis T. (1989). Molecular Cloning: A Laboratory Manual.

[44] Tsutakawa S.E., Classen S., Chapados B.R., Arvai A.S., Finger L.D., Guenther G., Tomlinson C.G., Thompson P., Sarker A.H., Shen B. (2011). Human flap endonuclease structures, DNA double-base flipping, and a unified understanding of the FEN1 superfamily. Cell.

[45] Udensi U.K., Tchounwou P.B. (2017). Potassium Homeostasis, Oxidative Stress, and Human Disease. Int. J. Clin. Exp. Physiol..

[46] Zacchia M., Abategiovanni M.L., Stratigis S., Capasso G. (2016). Potassium: From Physiology to Clinical Implications. Kidney Dis..

[47] Song B., Hamdan S.M., Hingorani M.M. (2018). Positioning the 5′-flap junction in the active site controls the rate of flap endonuclease-1-catalyzed DNA cleavage. J. Biol. Chem..

[48] Bornarth C.J., Ranalli T.A., Henricksen L.A., Wahl A.F., Bambara R.A. (1999). Effect of flap modifications on human FEN1 cleavage. Biochemistry.

[49] Gloor J.W., Balakrishnan L., Bambara R.A. (2010). Flap endonuclease 1 mechanism analysis indicates flap base binding prior to threading. J. Biol. Chem..

[50] Kaiser M., Lyamicheva N., Ma W., Miller C., Neri B., Fors L., Lyamichev V. (1999). A comparison of eubacterial and archaeal structure-specific 50 -exonucleases. J. Biol. Chem..

[51] Lyamichev V., Brow M.A., Varvel V.E., Dahlberg J.E. (1999). Comparison of the 5′ nuclease activities of taq DNA polymerase and its isolated nuclease domain. Proc. Natl. Acad. Sci. USA.

[52] Qiu J., Bimston D.N., Partikian A., Shen B. (2002). Arginine residues 47 and 70 of human flap endonuclease-1 are involved in DNA substrate interactions and cleavage site determination. J. Biol. Chem..

[53] Harrington J.J., Lieber M.R. (1994). The characterization of a mammalian DNA structure-specific endonuclease. EMBO J..

[54] Tsutakawa S.E., Thompson M.J., Arvai A.S., Neil A.J., Shaw S.J., Algasaier S.I., Kim J.C., Finger L.D., Jardine E., Gotham V.J.B. (2017). Phosphate steering by Flap Endonuclease 1 promotes 5′-flap specificity and incision to prevent genome instability. Nat. Commun..

[55] Bagur R., Hajnóczky G. (2017). Intracellular Ca^2+^ Sensing: Its Role in Calcium Homeostasis and Signaling. Mol. Cell.

[56] Krasikova Y., Rechkunova N., Lavrik O. (2021). Nucleotide Excision Repair: From Molecular Defects to Neurological Abnormalities. Int. J. Mol. Sci..

[57] Reardon J.T., Sancar A. (2003). Recognition and repair of the cyclobutane thymine dimer, a major cause of skin cancers, by the human excision nuclease. Genes. Dev..

[58] Kesseler K.J., Kaufmann W.K., Reardon J.T., Elston T.C., Sancar A. (2007). A mathematical model for human nucleotide excision repair: Damage recognition by random order assembly and kinetic proofreading. J. Theor. Biol..

[59] Park C.J., Choi B.S. (2006). The protein shuffle. Sequential interactions among components of the human nucleotide excision repair pathway. FEBS J..

[60] Zheng L., Jia J., Finger L.D., Guo Z., Zer C., Shen B. (2011). Functional regulation of FEN1 nuclease and its link to cancer. Nucleic Acids Res..

[61] Grasby J.A., Finger L.D., Tsutakawa S.E., Atack J.M., Tainer J.A. (2012). Unpairing and gating: Sequence-independent substrate recognition by FEN superfamily nucleases. Trends Biochem. Sci..

[62] Spiro C., Pelletier R., Rolfsmeier M.L., Dixon M.J., Lahue R.S., Gupta G., Park M.S., Chen X., Mariappan S.V., McMurray C.T. (1999). Inhibition of FEN-1 processing by DNA secondary structure at trinucleotide repeats. Mol. Cell.

[63] Zheng L., Zhou M., Chai Q., Parrish J., Xue D., Patrick S.M., Turchi J.J., Yannone S.M., Chen D., Shen B. (2005). Novel function of the flap endonuclease 1 complex in processing stalled DNA replication forks. EMBO Rep..

[64] Yang J., Freudenreich C.H. (2007). Haploinsufficiency of yeast FEN1 causes instability of expanded CAG/CTG tracts in a length-dependent manner. Gene.

[65] Beaver J.M., Lai Y., Rolle S.J., Liu Y. (2016). Proliferating cell nuclear antigen prevents trinucleotide repeat expansions by promoting repeat deletion and hairpin removal. DNA Repair.

[66] Sampathi S., Bhusari A., Shen B., Chai W. (2009). Human flap endonuclease I is in complex with telomerase and is required for telomerase-mediated telomere maintenance. J. Biol. Chem..

[67] Saharia A., Teasley D.C., Duxin J.P., Dao B., Chiappinelli K.B., Stewart S.A. (2010). FEN1 ensures telomere stability by facilitating replication fork re-initiation. J. Biol. Chem..

[68] Vallur A.C., Maizels N. (2010). Complementary roles for exonuclease 1 and flap endonuclease 1 in maintenance of triplet repeats. J. Biol. Chem..

[69] Teasley D.C., Parajuli S., Nguyen M., Moore H.R., Alspach E., Lock Y.J., Honaker Y., Saharia A., Piwnica-Worms H., Stewart S.A. (2015). Flap Endonuclease 1 Limits Telomere Fragility on the Leading Strand. J. Biol. Chem..

[70] Kokic G., Chernev A., Tegunov D., Dienemann C., Urlaub H., Cramer P. (2019). Structural basis of TFIIH activation for nucleotide excision repair. Nat. Commun..

[71] Lian F.M., Yang X., Jiang Y.L., Yang F., Li C., Yang W., Qian C. (2020). New structural insights into the recognition of undamaged splayed-arm DNA with a single pair of non-complementary nucleotides by human nucleotide excision repair protein XPA. Int. J. Biol. Macromol..

[72] Matsuda T., Saijo M., Kuraoka I., Kobayashi T., Nakatsu Y., Nagai A., Enjoji T., Masutani C., Sugasawa K., Hanaoka F. (1995). DNA repair protein XPA binds replication protein A (RPA). J. Biol. Chem..

[73] Wang M., Mahrenholz A., Lee S.H. (2000). RPA stabilizes the XPA-damaged DNA complex through protein-protein interaction. Biochemistry.

[74] Yang Z.G., Liu Y., Mao L.Y., Zhang J.T., Zou Y. (2002). Dimerization of human XPA and formation of XPA(2)-RPA protein complex. Biochemistry.

[75] Spegg V., Panagopoulos A., Stout M., Krishnan A., Reginato G., Imhof R., Roschitzki B., Cejka P., Altmeyer M. (2023). Phase separation properties of RPA combine high-affinity ssDNA binding with dynamic condensate functions at telomeres. Nat. Struct. Mol. Biol..

[76] Hanawalt P.C., Spivak G. (2008). Transcription-coupled DNA repair: Two decades of progress and surprises. Nat. Rev. Mol. Cell Biol..

[77] Son K., Schärer O.D. (2020). Repair, Removal, and Shutdown: It All Hinges on RNA Polymerase II Ubiquitylation. Cell.

[78] Belotserkovskii B.P., Tornaletti S., D’Souza A.D., Hanawalt P.C. (2018). R-loop generation during transcription: Formation, processing and cellular outcomes. DNA Repair.

[79] Sollier J., Stork C.T., García-Rubio M.L., Paulsen R.D., Aguilera A., Cimprich K.A. (2014). Transcription-coupled nucleotide excision repair factors promote R-loop-induced genome instability. Mol. Cell.

