# Supplementary material for: Does the XPA–FEN1 Interaction Concern to Nucleotide Excision Repair or Beyond?
Source: Biomolecules. 2024 Jul 9;14(7):814. doi: 10.3390/biom14070814 (PMC11274875; doi:10.3390/biom14070814)
Supplement: Supplementary file 1 [file biomolecules-14-00814-s001.zip › biomolecules-2980681-supplementary-update.pdf]

SUPPLEMENTAL INFORMATION

Does the XPA–FEN1 Interaction Concern to Nucleotide Excision Repair or Beyond?

Table S1. **Oligonucleotide sequences**

**Name**	**Sequences 5′ → 3′**
**60**	Ctatggcgaggcgattatcaacccatttagtcgtaatagtgaagagtcacgacaacatcg
**40**	Cgatgttgtcgtgactcttcactattacgactaaatgggt
**33**	Cgatgttgtcgtgactcttcactattacgacta
**17**	Cgatgttgtcgtgactc
**17up**	Ctatggcgaggcgatta
**B**	ctatggcgaggcgattatcaacccattgcagtgggtcttccgaacgac
**Fg**	gtcgttcggaagaccctgacgt**F**gcccaacttaatcgcctcgccatag
**48F**	Gtcgttcggaagaccctgacgttgcccaacttaatcgcctcgccatag-**FAM**

The modification is indicated as follows: **F** – Flu-dUMP (fluorescein dUMP derivative (5-{3-[6-(carboxyamido-fluoresceinyl)amidocapromoyl]allyl}-dUMP)). Cyan color highlights the bubble area and underlining indicate the 5'-flap sequence. We have used these oligonucleotides previously [Krasikova Y.S. et al., 2010; Krasikova Y.S. et al., 2018]. The sequence of oligonucleotides were chosen randomly and checked for ability to form non aimed DNA structures.

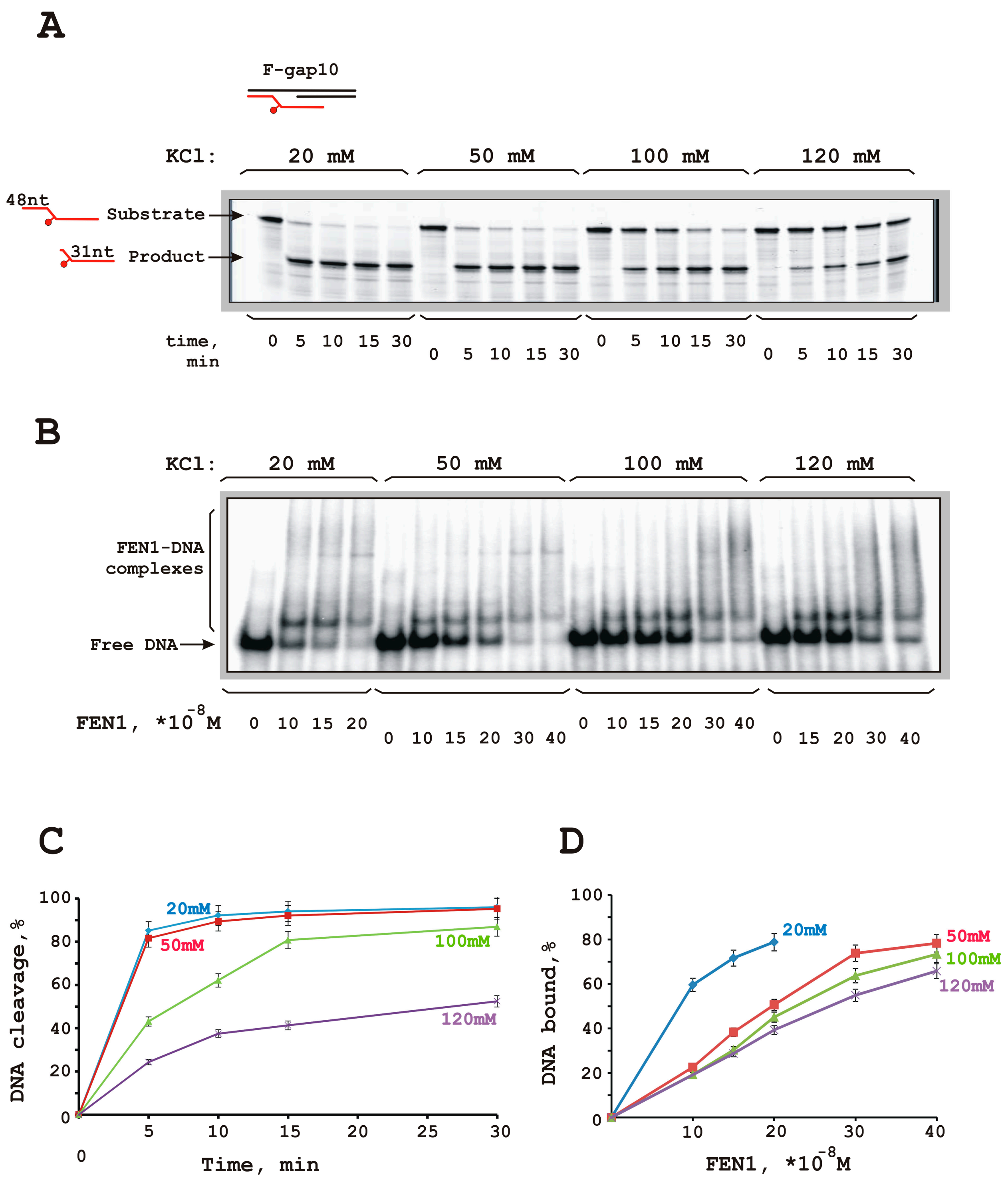

Figure S1. The monovalent ions inhibit FEN1-catalyzed DNA hydrolysis (**A**) especially in the range higher than 50 mM KCl (**C**). The KCl presence at the physiological concentration reduces FEN1 DNA binding affinity (**B**) and (**D**). The endonuclease activity of FEN1 was assessed on a 10nt gap containing substrate. Reaction mixtures (20 µL) contained FEN1 (10 nM), DNA substrate (10 nM), 5 mM Mg^{2+} (for nuclease experiments), 50 mM Tris-HCl pH 7.5, 1 mM dithiothreitol, at least 0.6 mg/ml BSA, KCl at the indicated concentrations and reactions were performed as described in Experimental Procedures. Substrate and cleavage product sizes are as indicated. Schematic representation of the DNA substrate is depicted above the figure. DNA binding was analyzed on the same substrate.

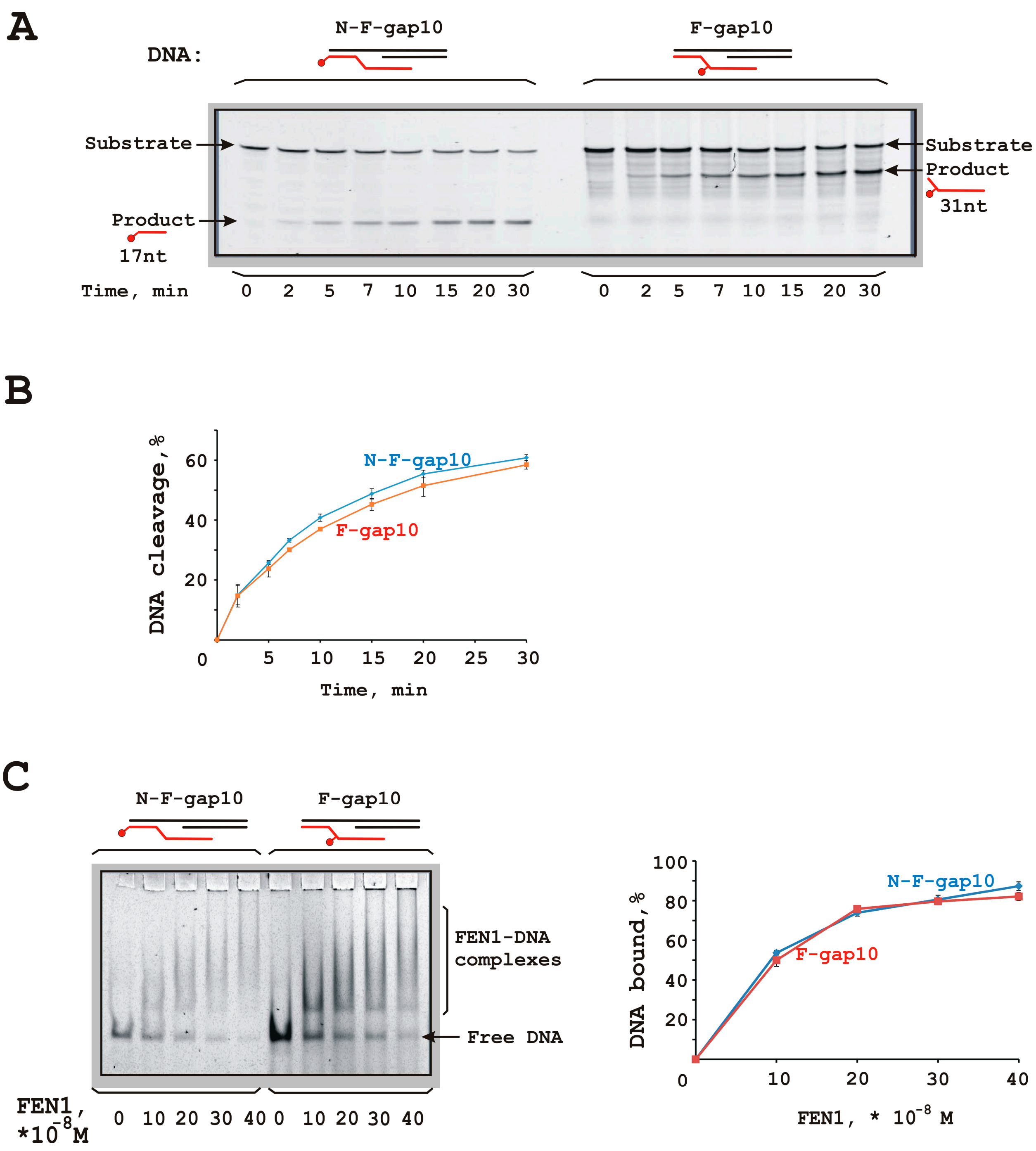

Figure S2. The fluorescein substitution does not affect FEN1 cleavage efficiency (**A** and **B**) and binding affinity (**C**). All experiments were performed with DNA containing 10nt gap. The oligonucleotide composition of DNA structure with undamaged flap is (60+33up+48F).
Binding experiments were also performed with 5 mM Ca^{2+} (**C**). Schematic representations of the DNA substrates are depicted above the figure.
(**A**) Reaction mixtures (20 µL) contained FEN1 (5 nM), DNA substrate (10 nM), 5 mM Mg^{2+}, 50 mM Tris-HCl pH 7.5, 1 mM dithiothreitol, 0.6 mg/ml BSA, 100 mM KCl. Reactions were performed as described in Experimental Procedures. Substrate and cleavage product sizes are as indicated. DNA binding was analyzed on the same substrate. (C) Reaction mixtures (10 µL) contained DNA substrate (10 nM), 0.1 nM pUC19 plasmid, 50 mM Tris-HCl pH 7.5, 100 mM KCl, 5 mM Ca^{2+}, 1 mM dithiothreitol, 0.6 mg/ml BSA, FEN1 at the indicated concentrations and reactions were performed as described in Experimental Procedures.

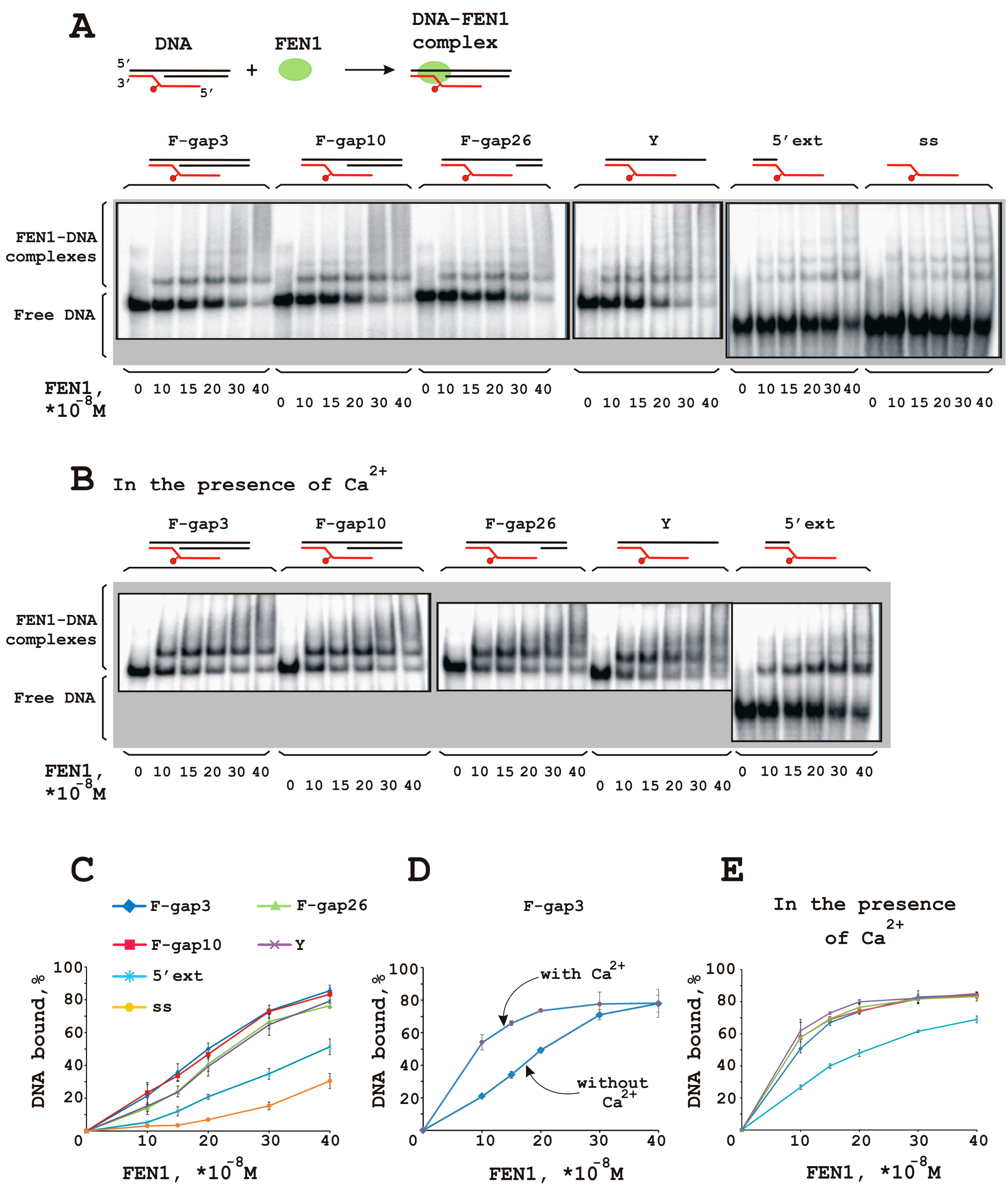

Figure S3. The gap size does not affect FEN1 binding affinity (**A**) and (**C**) but the presence of both template strand parts forming flap basis are indispensable for correct FEN1-DNA interaction. The number and intensity of product bands for 5′ext and ss DNA structures are nearly equal thus we assume same pattern of protein-DNA interaction. Binding experiments also performed with Ca2+ (**B**) and (**E**). FEN1 shows higher affinity in the calcium presence compare to divalent metal absence condition (**D**). Schematic representations of the DNA substrates are depicted above the figure.

Reaction mixtures (10 μL) contained DNA substrate (10 nM), 0.1 nM pUC19 plasmid, 50 mM Tris-HCl pH 7.5, 100 mM KCl, 5 mM Ca^{2+} (in the case of calcium experiments), 1 mM dithiothreitol, at least 0.6 mg/ml BSA, FEN1 at the indicated concentrations and reactions were performed as described in Experimental Procedures.

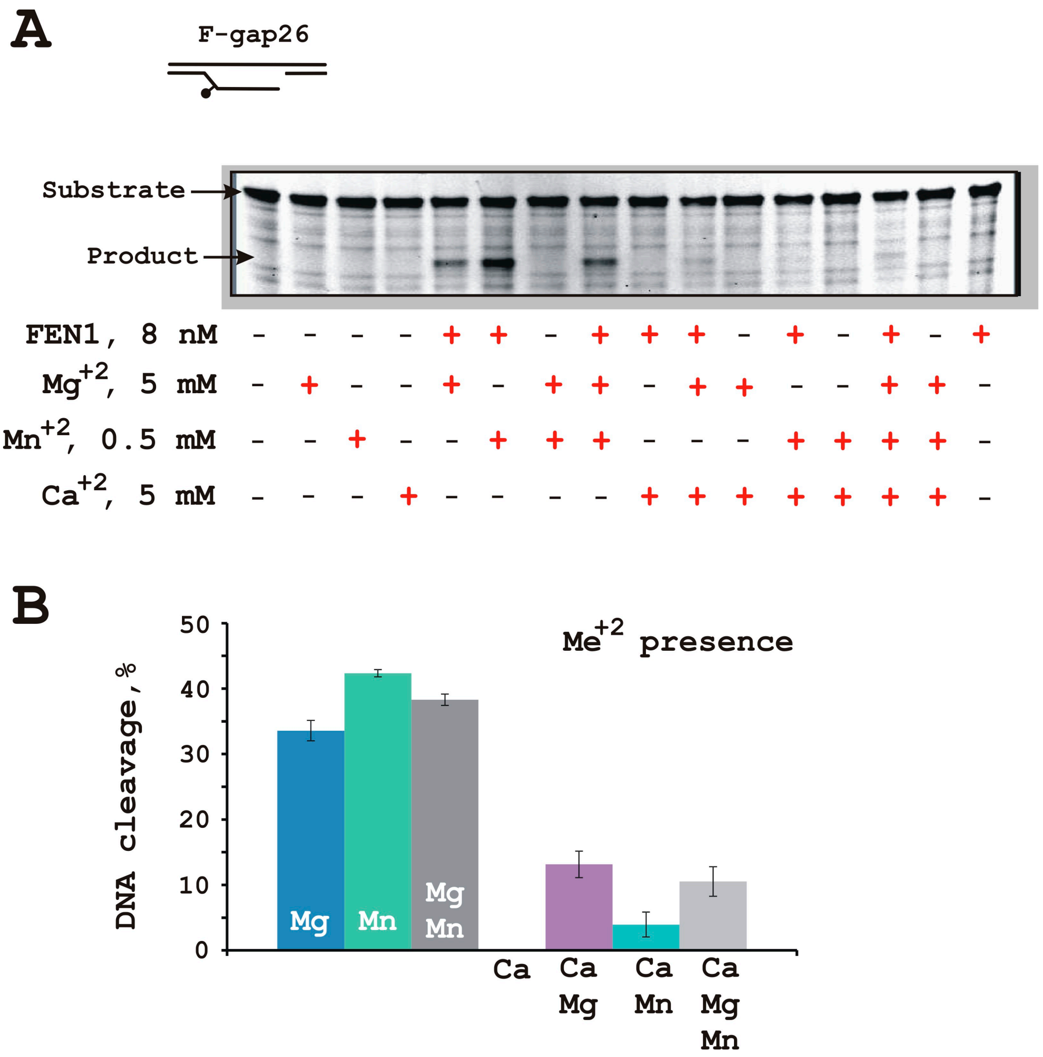

Figure S4. The divalent metal ions affect differently on FEN1 nuclease activity (**A**). Substitution of 5 mM $MgCl_2$ with 0.5 mM $MnCl_2$ or combination of these metals lead to higher activity. Replacement of 5 mM MgCl2 with 5 mM $CaCl_2$ completely inhibited the reaction. In a mixing reactions, $CaCl_2$ with $MgCl_2$ and $MnCl_2$ presence resulted in great inhibition of FEN1 activity (**B**).

Reaction mixtures (20 µL) contained FEN1 (10 nM), DNA substrate (10 nM), 50 mM Tris-HCl pH 7.5, 100 mM KCl, 1 mM dithiothreitol, at least 0.6 mg/ml BSA, divalent metals at the indicated concentrations and reactions were performed as described in Experimental Procedures.

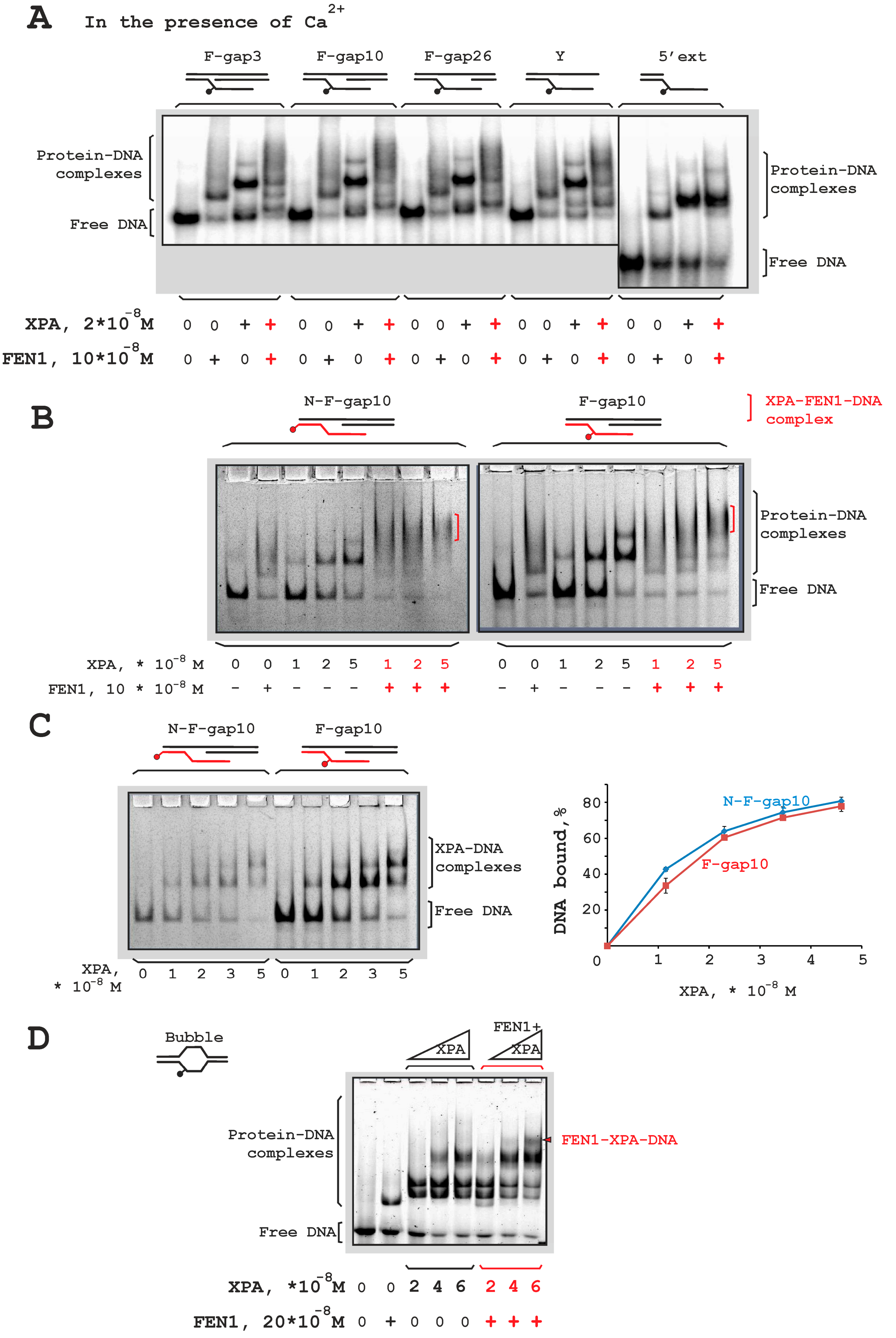

Figure S5. FEN1-XPA-DNA complex is formed equally with different sizes gap DNAs in the presence of 5 mM $CaCl_2$ (**A**). The FEN1-XPA-DNA complex is formed equally with damaged and undamaged flap structures (**B**) in the presence of 5 mM Ca^{2+}. The oligonucleotide composition of DNA structure with undamaged flap is (60+33up+48F). XPA alone also binds to

damaged and undamaged flaps equally in the presence of 5 mM Mg^{2+} (**C**). The ternary complex is also registered with bubbled DNA (bubbled DNA oligonucleotides composition is B+Fg) mimicking NER pre-incision complex (**D**).
Reaction mixtures (10 μL) contained DNA substrate (10 nM), 0.1 nM pUC19 plasmid, 50 mM Tris-HCl pH 7.5, 100 mM KCl, 5 mM Ca^{2+} (**A** and **B**) or Mg^{2+} (**C** and **D**), 1 mM dithiothreitol, at least 0.6 mg/ml BSA, FEN1 and/or XPA at the indicated concentrations and reactions were performed as described in Experimental Procedures.

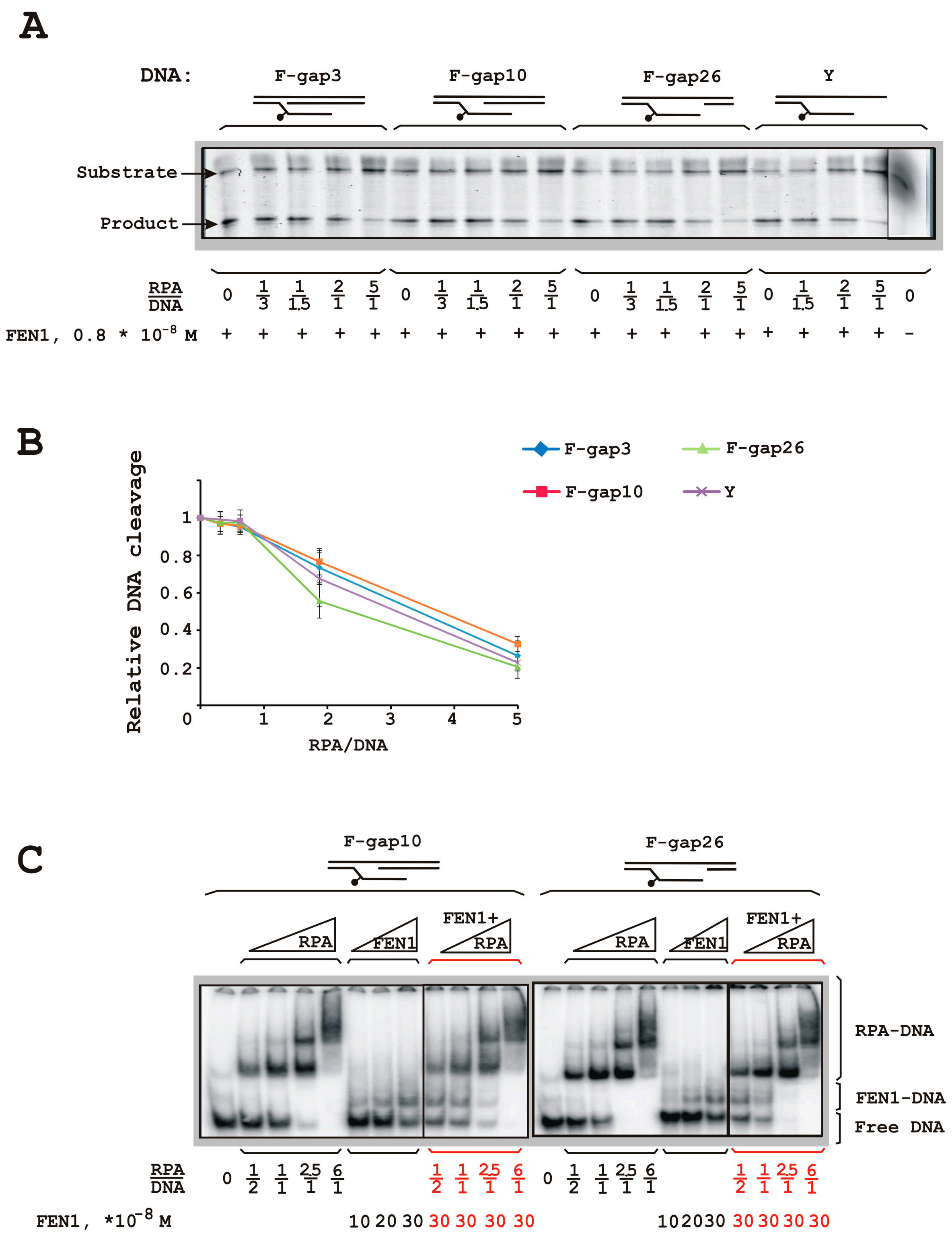

Figure S6. RPA competes with FEN1 for binding (**A**) and displace it from DNA (FEN1-DNA complex titration by the increasing amount of RPA was resulted in FEN1 complex disappearance (**C**)). RPA binding position (inside the gap platform [Krasikova YS et al., 2018]) also inhibits FEN1 cleavage, especially in the case of F-gap26 structure (**B**).

Reaction mixtures (20 µL) contained DNA substrate (10 nM), 50 mM Tris-HCl pH 7.5, 100 mM KCl, 5 mM Mg^{2+} (in the case of cleavage experiments), 1 mM dithiothreitol, at least 0.6 mg/ml BSA, FEN1 and/or RPA at the indicated concentrations and were incubated at 37 °C for 10 min. For details, see Experimental Procedures.

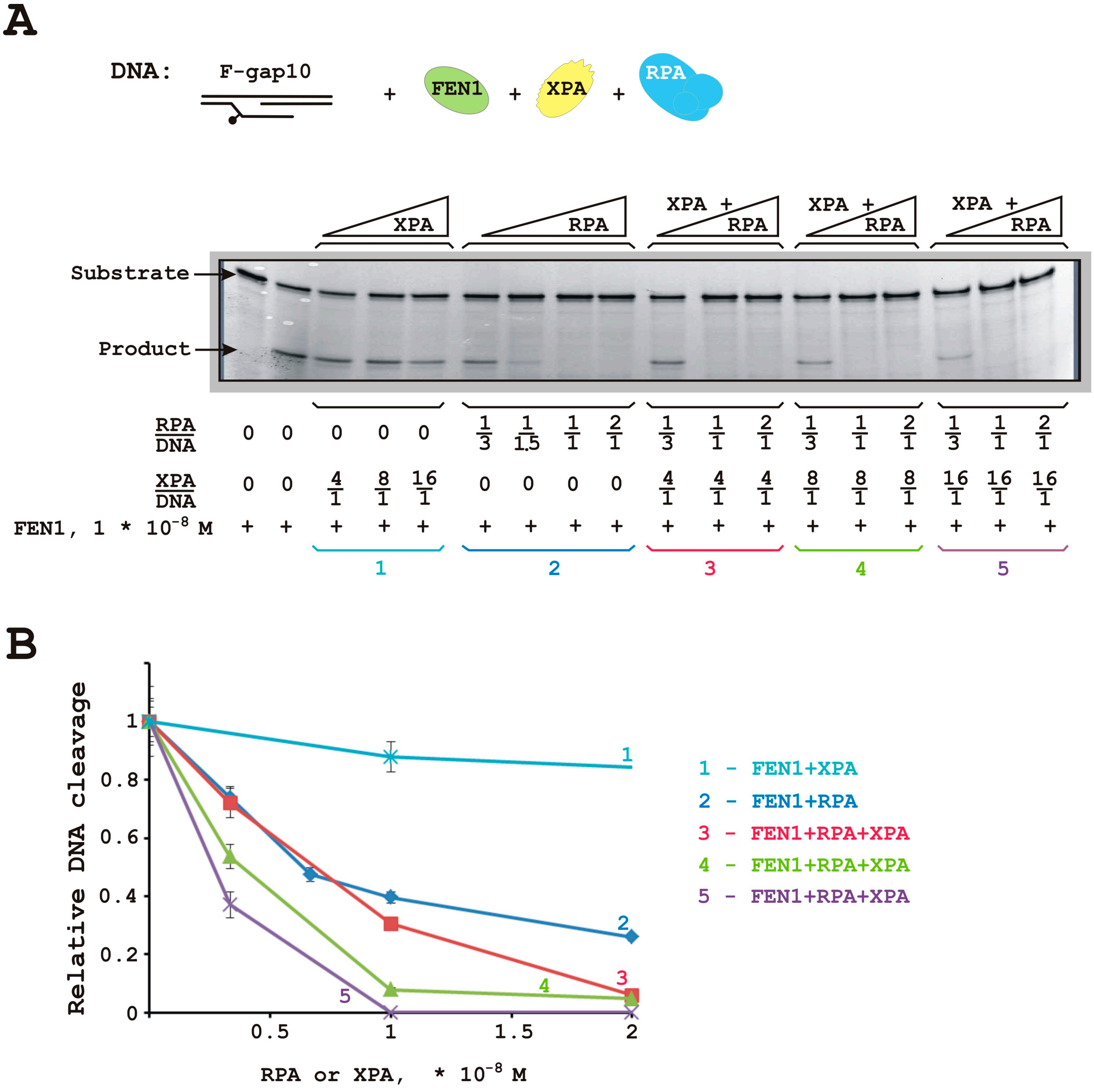

Figure S7. Inhibition of FEN1 catalytic activity by RPA is classical for displacement by DNA binding protein (**A**). At the same time, XPA inhibits FEN1 activity mildly which cannot be interpreted as competitive inhibition. Even under high XPA excess, product bands did not disappear. Under conditions of simultaneous presence of RPA and XPA in the reaction mixture, the inhibition profile matched the one for RPA alone (**B**).

Reaction mixtures (20 µL) contained DNA substrate (10 nM), 50 mM Tris-HCl pH 7.5, 100 mM KCl, 5 mM Mg^{2+}, 1 mM dithiothreitol, at least 0.6 mg/ml BSA, FEN1 and/or RPA with or without XPA at the indicated concentrations and were incubated at 37 °C for 5 min. For details, please see Experimental Procedures.

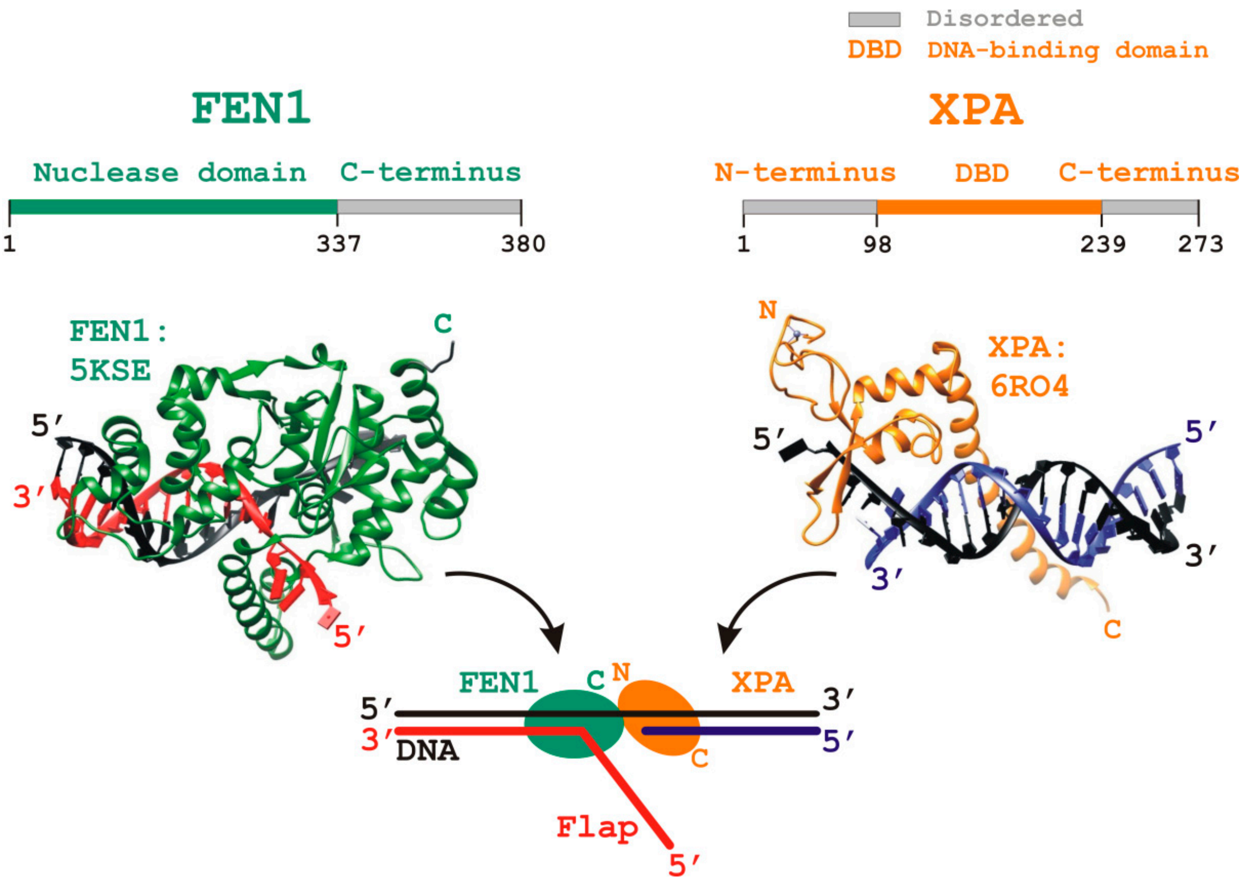

Figure S8. Spatial organization of the putative FEN1–XPA–DNA complex on the flapped DNA. FEN1 recognizes 5′-flap DNA by contacting both sides of the flap basis: the dsDNA and ssDNA template inside the gap (X-ray structure PDB ID: 5KSE). XPA is forced to bind to the opposite ss–dsDNA junction with a 3′-extention (cryo-EM structure PDB ID: 6RO4). Assuming such FEN1 and XPA locations on DNA, we suggest that these protein–protein interactions are formed by the C-terminus (aa 337–380) of FEN1 and the N-terminus (aa 1–98) of XPA (these regions are highlighted in grey). FEN-1 consists of a nuclease core domain harboring catalytic activity and a C-terminal extension that may be important for protein–protein interactions. In the absence of a protein partner, the extended C-terminus is predicted to be intrinsically disordered [Zheng L. et al., 2011]. Several FEN1 interaction partners (e.g., PCNA, WRN, APE1, EndoG, Rad9–Rad1–Hus1, and p300) probably utilize this region [Sakurai S. et al., 2005]. The structures were generated in the UCSF Chimera software (version 1.16 [build 42360]).

Krasikova, Y.S.; Rechkunova, N.I.; Maltseva, E.A.; Petruseva, I.O.; Lavrik, O.I. Localization of xeroderma pigmentosum group A protein and replication protein A on damaged DNA in nucleotide excision repair. *Nucleic Acids Res*. **2010**, 38, 8083–8094. https://doi.org/10.1093/nar/gkq649.

Krasikova, Y.S.; Rechkunova, N.I.; Maltseva, E.A.; Lavrik, O.I. RPA and XPA interaction with DNA structures mimicking intermediates of the late stages in nucleotide excision repair. *PLoS ONE* **2018**, 13, e0190782. https://doi.org/10.1371/journal.pone.0190782.

Sakurai, S.; Kitano, K.; Yamaguchi, H.; Hamada, K.; Okada, K.; Fukuda, K.; Uchida, M.; Ohtsuka, E.; Morioka, H.; Hakoshima, T. Structural basis for recruitment of human flap endonuclease 1 to PCNA. *EMBO J.* **2005**, *24*, 683-93. doi: 10.1038/sj.emboj.7600519.

Zheng, L., Jia, J., Finger, L.D., Guo, Z., Zer, C., Shen, B. Functional regulation of FEN1 nuclease and its link to cancer. *Nucleic Acids Res.* **2011**, *39*, 781-94. doi: 10.1093/nar/gkq884.
